# Supplementary material for: Thermodynamic Insights of the Molecular Interactions of Dopamine (Neurotransmitter) with Anionic Surfactant in Non-Aqueous Media
Source: Pharmaceuticals (Basel). 2023 Aug 22;16(9):1187. doi: 10.3390/ph16091187 (PMC10537161; doi:10.3390/ph16091187)
Supplement: Supplementary file 1 [file pharmaceuticals-16-01187-s001.zip › pharmaceuticals-2487279-supplementary.pdf]

Supporting Information for publication

Thermodynamic Insights of the Molecular Interactions of Dopamine (Neurotransmitter) with Anionic Surfactant in Non-Aqueous Media

Arshid Nabi [1,*], Christopher G. Jesudason, Jamal S. M. Sabir [2,3] and Majid Rasool Kamli [2,]

1. Tables (S1–S3)

Table S1

Values of experimental conductivities (κ^b/ $\mu S\cdot cm^{-1}$) of sodium dodecyl sulfate (SDS) in 0.05 m (*mol/kg*$^{-1}$) of dopamine hydrochloride (DA) in ethanol (Et-OH) solutions from T^c = (298.15 to 313.15) K at Pressure (p)^d = 0.1 MPa.

	κ /$mS\cdot cm^{-1}$			
m^a / mol·kg^{-1}	298.15	303.15	308.15	313.15
0.001	1.20	1.70	2.29	3.07
0.002	3.21	4.08	4.76	5.55
0.003	5.55	6.37	7.05	8.04
0.004	7.72	8.76	9.44	10.32
0.005	9.93	11.07	11.83	12.71
0.006	12.00	13.35	14.12	15.08
0.007	14.22	15.64	16.59	17.46
0.008	16.50	17.93	18.97	19.73
0.009	17.55	19.17	20.58	21.53
0.010	18.59	20.11	21.44	22.78
0.011	19.55	21.09	22.50	23.74
0.012	20.29	22.05	23.39	24.91

^{a}m is the molality of SDS in (ethanol + DA) system.

Standard uncertainties, u, are u(T)^c = ±0.01 K, u(p)^d = ±0.002 MPa, and $u_r(\kappa)^b$ = ±0.015 mS·cm^{-1} (level of confidence = 0.68).

Table S2

Values of experimental densities (ρ^b/g cm^{-3}) of sodium dodecyl sulfate (SDS) in 0.05 m (mol/kg^{-1}) of dopamine hydrochloride (DA) in ethanol (Et-OH) solutions from u(T^c)= (298.15 to 313.15) K at Pressure $(p)^d$ = 0.1 MPa.

	ρ /g cm^{-3}			
$m_T{}^a$ / mol·kg^{-1}	298.15	303.15	308.15	313.15
0.001	0.7978	0.7972	0.7965	0.7957
0.002	0.7972	0.7965	0.7957	0.7949
0.003	0.7965	0.7958	0.7948	0.7938
0.004	0.7959	0.7950	0.7940	0.7929
0.005	0.7952	0.7943	0.7931	0.7920
0.006	0.7944	0.7934	0.7922	0.7910
0.007	0.7937	0.7927	0.7913	0.7901
0.008	0.7930	0.7919	0.7905	0.7890
0.009	0.7913	0.7902	0.7887	0.7877
0.010	0.7894	0.7880	0.7867	0.7852
0.011	0.7873	0.7859	0.7844	0.7830
0.012	0.7852	0.7838	0.7823	0.7807

[a]m is the molality of SDS in (ethanol + DA) system.

Standard uncertainties, u, are $u(T)^c$ = ±0.01 K, $u(p)^d$ = ±0.002 MPa, and $u_r(\rho)^b$ = ±0.001 g .cm^{-3} (level of confidence = 0.68).

Table S3

Values of experimental surface tension (γ^{b}/ mN.m^{-1}) of sodium dodecyl sulfate (SDS) in 0.05 m (mol/kg^{-1}) of dopamine hydrochloride (DA) in ethanol (Et-OH) solutions from T^{c} = (298.15 to 313.15) K at Pressure $(p)^{d}$ = 0.1 MPa.

	γ / mN.m^{-1}			
$m_T{}^{a}$ / mol·kg^{-1}				
0.001	39.63	38.21	36.75	35.13
0.002	37.28	36.00	34.46	33.29
0.003	35.23	33.88	32.54	31.06
0.004	33.08	31.91	30.65	29.41
0.005	30.98	29.63	28.64	27.67
0.006	28.83	27.61	26.62	25.51
0.007	26.71	25.54	24.60	23.71
0.008	24.46	23.48	22.74	21.94
0.009	23.46	22.43	21.46	20.60
0.010	22.67	21.93	20.90	20.17
0.011	21.98	21.29	20.62	19.94
0.012	21.55	20.86	20.09	19.57

[a]m is the molality of SDS in (ethanol + DA) system.

Standard uncertainties, u, are $u(T)^{c}$ = ±0.01 K, $u(p)^{d}$ = ±0.002 MPa, and $u_r(\gamma)^{b}$ = ±0.3 mN.m^{-1} (level of confidence = 0.68).
